# What is the impact of infrapopliteal endovascular intervention on free flap survival in diabetic foot reconstruction?

**DOI:** 10.1186/s13018-020-02173-9

**Published:** 2021-01-11

**Authors:** Duy Quang Thai, Dong Hwan Lee, Woo Beom Lee, Hyung Min Hahn, Il Jae Lee

**Affiliations:** Department of Plastic and Reconstructive Surgery, Ajou University School of Medicine, 164 World cup-ro, Yeongtong-gu, Suwon-si, Gyeonggi-do 16499 Republic of Korea

**Keywords:** Diabetic foot, Peripheral arterial disease, Endovascular intervention, Percutaneous transluminal Angioplasty, Free flap

## Abstract

**Background:**

The combination of endovascular intervention and microvascular free flap transfer has been effectively used for chronic ischemic wounds of lower limb. The aim of this study was to determine the influence of angioplasty on free flap survival in diabetic foot ulcer reconstruction.

**Methods:**

A retrospective research was conducted for 46 diabetic patients with chronic ulcer of the foot. All patients underwent free flap reconstruction because of their non-healing wound with tendon or bone exposure. Patient’s demography, clinical data related to vascular status, vascular intervention, and free flap transfer procedure were collected. Flap survival rate was compared between the group with severe arterial stenosis group and non-severe stenosis group. It was also compared among groups with different revascularization results.

**Results:**

The average age of patients was 56.2 ± 10.8 years. There were 14 (30.4%) men and 32 (69.6%) women. Of 46 patients, 23 (50%) had severe infrapopliteal arterial stenosis. All 23 patients underwent endovascular intervention. Their final results of the pedal arch were type 1 in 13 patients, type 2A in 7 patients, type 2B in 2 patients, and type 3 in 1 patient. Total flap necrosis was found in 5 (10.9%) cases, marginal necrosis in 4 (8.7%) cases, and wound dehiscence in 4 (8.7%) cases. There was no significant difference in flap loss between severe arterial stenosis patients and non-severe arterial stenosis patients. In the severe arterial stenosis group, after endovascular intervention, patients with type 1 of pedal arch had a significantly lower rate of total flap necrosis than others. There was no association between the use of revascularized recipient artery and flap survival.

**Conclusions:**

Our study revealed that the quality of pedal arch was crucial for free flap survival. Thus, PTA should aim to re-establish a complete pedal arch to increase wound healing rate and flap success.

## Background

Diabetic foot ulcer as a consequence of neuropathy and peripheral arterial disease (PAD) is a common complication of diabetes mellitus (DM). Although most diabetic defects could be treated with conservative modalities or simple debridement and suture, a broad and deep ulcer with bone and tendon exposure is more challenging. The extensive diabetic foot ulcer with PAD often leads to major amputation that not only reduces the quality of life but also increase the mortality rate [1]. Some studies have successfully employed local flap or skin graft; however, generally salvaging these ischemic limbs without a strong and reliable flap is limited [2, 3]. In 1985, Brigg et al. first combined vascular bypass surgery and free tissue transfer to reconstruct an ischemic lower limb [4]. In this combination, arterial bypass surgery played a crucial role to increase blood supply for distal limbs and facilitate free flap transfer as well. However, after endovascular intervention was introduced with several advantages, it has replaced the bypass surgery to be the primary indication in critical limb ischemia management [5]. Subsequently, numerous studies have investigated the combination of preoperative angioplasty and microvascular free flap surgery. However, the influence of percutaneous transluminal angioplasty (PTA) on free flap success in diabetic patients remains unclear. Therefore, we reviewed our patients who underwent microvascular free flap reconstruction because of diabetic foot ulcers. Our purpose was to determine the impact of endovascular revascularization on the success of free flap and to explore the other risk factors of microvascular surgery after angioplasty.

## Methods

A retrospective study was carried out on all diabetic patients who underwent chronic foot ulcers that were reconstructed with free flap transfer in Plastic and Reconstructive Department of Ajou University Hospital from 2011 to 2019. Free flap was indicated for patients because they had soft tissue defects with exposure of bones and/or tendons that could not be treated by other simple procedures. We excluded patients who suffered from acute wounds such as trauma or tumor resection. Patients were divided into two groups based on their vascular status: severe PAD group with grade 4 and 5 vascular stenosis (Table 1), and non-severe PAD group with others. All the patients of severe PAD group underwent PTA in at least one of three main arteries for increasing the distal perfusion for the foot via the pedal arch. Patients were followed up for more than 6 months.

**Table 1:** Grading of severity of arterial stenosis [6]

Grade	Criteria
0	Normal patency
1	Mild (stenosis of less than 25% of diameter)
2	Moderate (stenosis of 25–50% of diameter)
3	Moderate severe (stenosis of 50–75% of diameter)
4	Severe (stenosis of 75–99% of diameter)
5	Complete occlusion

Clinical data were collected via patients’ charts, radiographs of the lower extremity, preoperative photographs, and postoperative photographs. These data consisted age, gender, medical histories, wounds location, size of defects, vascular status, details of endovascular procedures, final result of pedal arch, flap of choice, flaps size, pedicle length, flap outcomes, complications, and follow-up information.

### Treatment procedure

Treatment started with careful assessment of the wound as well as the status of vessels. If there were any symptoms of infection such as redness, swelling, or hot skin, debridement would be indicated. Simultaneously, if we did not identify the distal pulsation of infragenicular arteries by palpation or handheld Doppler, patient would be further evaluated for vasculopathy and neuropathy. Patients would undergo pre-operative CT angiography for evaluation of infrapopliteal arterial condition. According to results of CT angiography, the severity of stenosis was graded as shown in Table 1. Patients with grade 4 and grade 5 of stenosed vessels would undergo conventional angiography, and PTA in at least one of three main arteries would be done if the state were confirmed to increase the distal perfusion for the foot via the pedal arch. Post-angioplasty pedal arch was classified as shown in Table 2. Free flap transfer surgery was performed once there was at least one patent blood flow to the foot and the recipient base was clean. All operations were decided and carried out by one senior surgeon.

**Table 2:** Classification of pedal arch [7]

Types	Blood supply
Type 1	From both DPA and plantar artery
Type 2A	Only from DPA
Type 2B	Only from plantar artery
Type 3	Both DPA and plantar arteries occluded

### Statistical analysis

Continuous variables were expressed as mean ± standard deviation. Chi-square test and Fisher’s exact test were employed to analyze categorical variables. Statistical significance was considered at *p* value < 0.05. All statistical analyses were performed with IBM SPSS 20.0.

## Results

A total of 46 foot-defects in 46 diabetic patients were covered with free flaps. Patient’s demographic characteristics are shown in Table 3. The average age of patients was 56.2 ± 10.8 years (range, 37 to 80 years). There were 14 (30.4%) men and 32 (69.6%) women. Of 46 patients, 8 (17.4%) were with end-stage renal disease (ESRD) and 17 (37%) were active smokers. The most common location was forefoot (*n* = 15), followed by heel and dorsum (*n* = 11 and *n* = 10, respectively) (Table 3). The mean size of defect was 10 cm × 6 cm (range, 3 cm × 2 cm to 20 cm × 18 cm).

**Table 3:** Patient’s demographic characteristics

Characteristic		No of patient (%)
Sex	Male	32 (69.6)
	Female	14 (30.4)
Location of defect	Forefoot	15 (32.6)
	Dorsum	10 (21.7)
	Heel	11 (23.9)
	Plantar	2 (4.3)
	Ankle	2 (4.3)
	Lateral malleolar	6 (13)
Comorbidity	ESRD	8 (17.4)
	Severe PAD	23 (50)
	Smoking	17 (36.9)
Infection		13 (28.3)

Twenty-three (50%) patients were diagnosed as severe PAD with grade 4 or 5 of below the knee arterial stenosis. Single-arterial stenosis was found in nine (9/23, 39.1%) patients. Double-arterial stenosis was found in seven (7/23, 30.4%) patients. Triple-arterial stenosis was also found in seven (7/23 (30.4%) patients. The mean interval between PTA procedure and free flap surgery was 15.43 ± 12.28 days, ranging from 4 to 54 days. Angioplasty failed in two arteries, one anterior tibial artery (ATA) in a single-arterial stenosis patient, and one posterior tibial artery (PTa) in a triple-arterial stenosis patient. Post-angioplasty pedal arch was type 1 in 13 (13/23, 56.5%) patients, type 2A in 7 (7/23) patients, type 2B in 2 (2/23) patients, and type 3 in one patient.

Two donor sites of free flap were anterolateral thigh (ALT) in 40 cases and medial sural artery perforator (MSAP) in 6 cases. The average size of flap was 12 cm × 7 cm (range, 5.5 cm × 3 cm to 20 cm × 12 cm). The mean pedicle length was 7.7 ± 1.9 cm (range, 4 cm to 12 cm). Recipient vessels were 13 PTa, 33 ATA, and its branch included dorsalis pedis artery (DPA) and the first dorsal metatarsal artery. Among them, 18 (39.1%) recipient arteries were revascularized vessels. End-to-side method was used for 40 (87%) flaps and end-to-end method was used for 6 (13%) flaps. Thirty-seven flaps had 2 veins and 9 flaps had 1 vein only (Table 4). Total flap necrosis was seen in five (10.9%) cases. Of these patients, two new ALT flaps were successfully replaced in two patients. Other patients were treated with additional debridement, negative pressure wound therapy, and split thickness skin graft. Other complications included marginal necrosis (4 flaps) and wound dehiscence (4 flaps) treated with simple closure or skin graft without any special event. During the perioperative period, there was no in-hospital mortality. However, two patients died at 5 months after operation due to pneumonia and at 7 months after operation due to urinary tract infection. No further major amputation was required during the follow-up period.

**Table 4:** Flap characteristics

Characteristic		No. of patients (%)
Type of flap	ALT	40 (87)
	MSAP	6 (13)
Recipient art	ATA	33 (71.7)
	PTa	13 (28.3)
Arteriorrhaphy	E-E	6 (13)
	E-S	40 (87)
Vein	1	9 (19.6)
	2	37 (80.4)
Complication	Total flap necrosis	5 (10.9%)
	Marginal necrosis	4 (8.7%)
	Wound dehiscence	4 (8.7%)

Four of five total necrosis flaps were found in patients with ESRD. There was a significant association between ESRD and flap failure (*p* = 0.002). There was no significant difference in flap loss rate between choices of flap, types of arteriorrhaphy, or the use of revascularized recipient artery. The rate of total flap necrosis in patients who underwent PTA procedure because of severe PAD was not significantly higher than in patients without severe PAD. In the severe PAD group (*n* = 23), the rate of flap loss was significantly different between type 1 of pedal arch patients and other types (*p* = 0.024). Among these patients, total flap necrosis was only found in patients with type 2 or 3 of the pedal arch (Table 5).

**Table 5:** Chi-square analysis for flap success

		Flap total necrosis	Flap success	***p***
ESRD	ESRD	4	4	0.002^*^
	No	1	37	
PAD	Severe PAD	4	19	0.346
	Non- severe PAD	1	22	
Recipient arteries	Revascularized	3	15	0.365
	Normal	2	26	
Post-angioplasty pedal arch (*n* = 23)	Type I	0	13	0.024^*^
	Types II and III	4	6	

*Statistically significant

Non-severe PAD = grade 0–3, severe PAD = grades 4 and 5

## Case reports

### Case 1

**Fig. 1:**
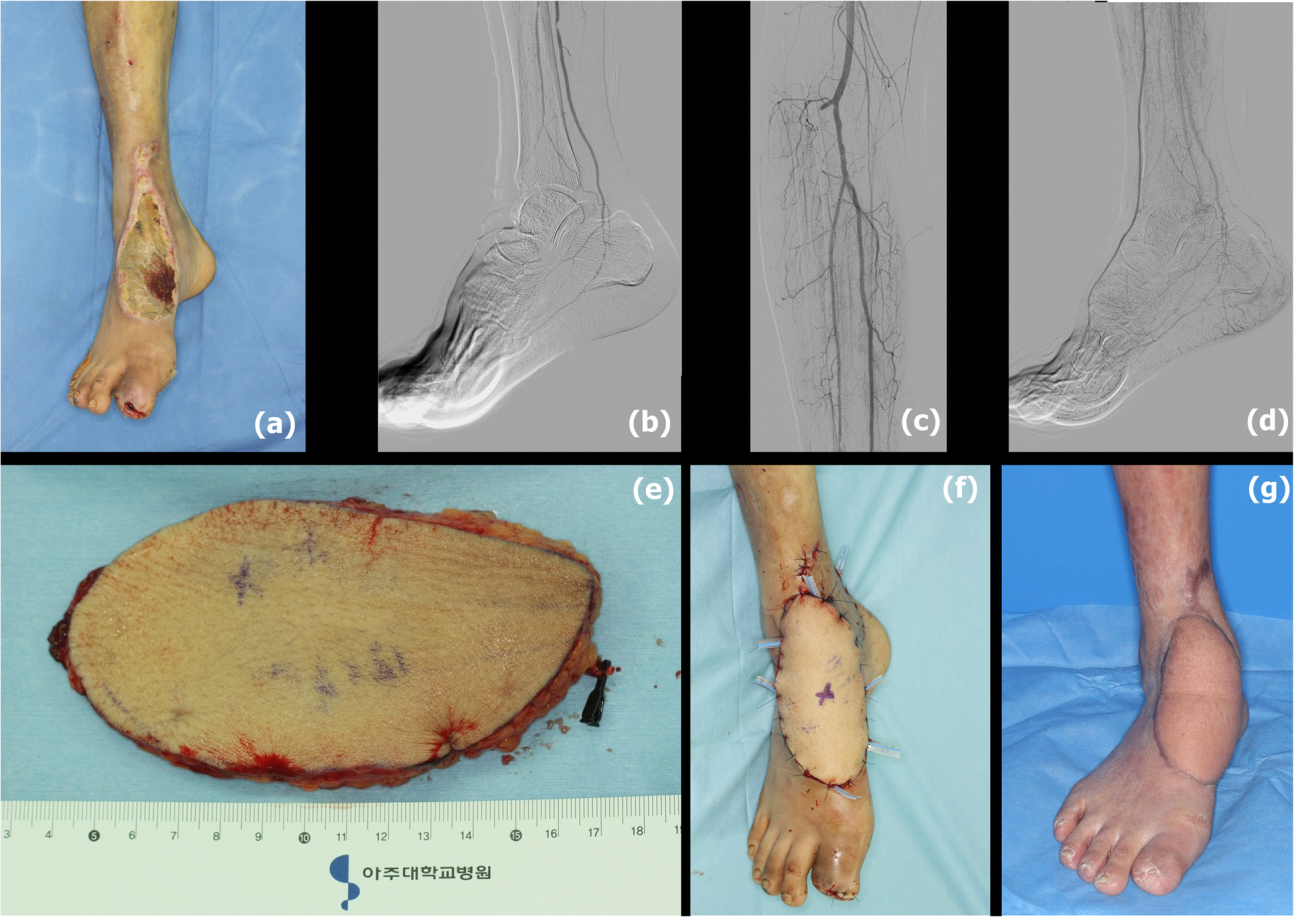
**a** A chronic wound of dorsal foot. **b**, **c** Anterior tibial artery was totally occluded, and pre-operative angiography indicated the type 2B of the pedal arch. **d** Anterior tibial artery was successfully revascularized and the foot was supplied by both dorsal pedis artery and plantar artery. **e** ALT free flap. **f** One day after operation. **g** Four months post-operation

A 53-year-old man with a 17-year history of diabetes and ESRD was admitted for an unhealed wound over the dorsal of right foot. Size of defect was 10 cm × 5 cm. His anterior tibial arterial pulse was not palpable in physical examination. CT angiogram revealed total occlusion of the ATA at the proximal level (Fig. 1). In angiography, the status of ATA was confirmed. Immediate balloon angioplasty was performed. ATA was successfully revascularized and complete blood flow in the pedal arch was resumed. After serial debridement, the tendon and bone were exposed. A 15 cm × 8 cm ALT flap with one perforator was used for soft-tissue coverage. The revascularized ATA was selected as the recipient artery and recipient veins were concomitant veins. Pedicle length was 7 cm. Arterial anastomosis and venous anastomosis were performed in end-to-side and end-to-end manners, respectively. No postoperative complications were noted. Four months of follow-up revealed a well-healing, well-perfused free flap. The patient was able to ambulate without complaints.

### Case 2

**Fig. 2:**
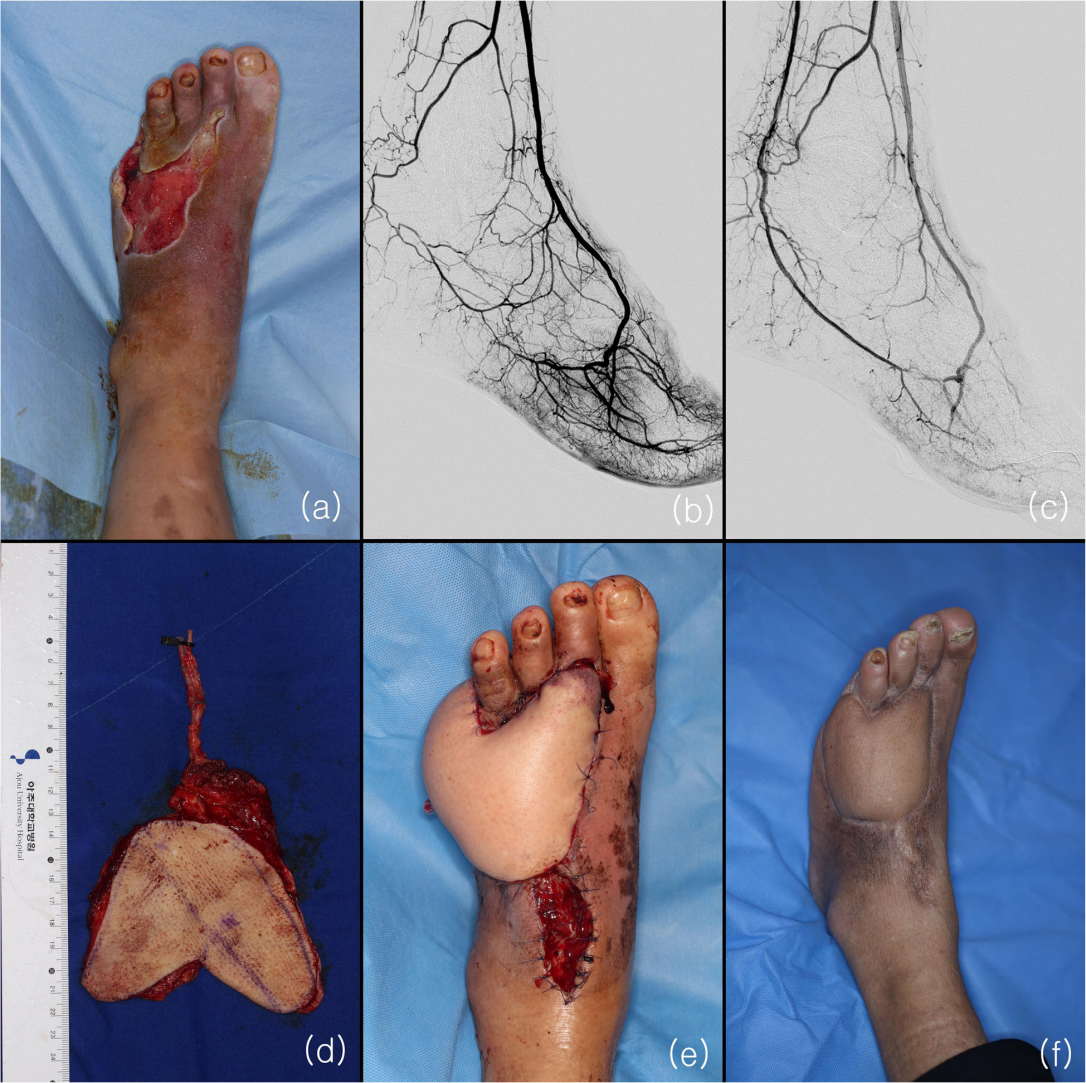
**a** Soft-tissue defect with 5th metatarsal bone exposure. **b** Angiography showing total occlusion of posterior tibial artery and plantar artery. **c** Complete pedal arch (type 1) was seen post-angioplasty. **d** A 13 × 9 cm ALT flap was performed. **e** Two days post-operation. **f** Four-year follow-up

A 55-year-old man with a 20-year history of diabetes presented with diabetic gangrene on his 5th toe. Serial debridement and amputation were performed and free tissue transfer was considered for covering the 5th metatarsal bone (Fig. 2). After debridement, the size of wound was 8 cm × 12 cm. Preoperative angioplasty indicated the absence of blood flow from the PTa to the pedal arch because of total occlusion. He underwent balloon angioplasty of the left PTa and plantar artery. After angioplasty, complete pedal arch was seen with the supply from both DPA and the plantar artery. Eight days after revascularization, an ALT flap was harvested and anastomosed to the ATA in an end-to-side manner. Size of flap was 9 cm × 13 cm and pedicle length was 6 cm. No additional surgery was required. Four years of follow-up revealed a well-healing, well-perfused free flap without another wound.

## Discussion

Although numerous studies have shown that extensive DM foot ulcer could be safely treated with free flap transfer, the presence of PAD in diabetic patient is always the challenging situation for the surgeon [8–11]. Oh et al. reported that patients with PAD had 17.59 times higher risk of flap failure [12]. In 1985, Briggs et al. firstly described the combination of vascular bypass surgery and free tissue transfer which expressively increased the salvage rate of the critical ischemic diabetic limb [4, 13–16]. Bypass surgery was successfully used for chronic limb ischemia management earlier on. However, PTA has slowly superseded open surgical bypass because of its advantages including lower complication and shorter length of hospitalization. It is also feasible for the elderly and patients with poor general condition, even in patients with severe multi-segmental occlusive disease [17–19]. In addition, with the introduction of small-scale balloon and drug-coated balloon, PTA is significantly effective with better primary patency and lower rate of restenosis [20]. Our institute has recently employed PTA as the first-line treatment for infrainguinal PAD patients. In this study, 23 patients were diagnosed with severe PAD. All of them underwent PTA before the flap transfer surgery to achieve the straight-line blood flow to the pedal arch. However, we failed to reconstruct the pedal arch in one patient classified as type 3. Despite having no impact on amputation rate, the quality of pedal arch can affect the rate of wound healing and time to healing as well [7, 21]. In this series, we also found that the quality of pedal arch had a crucial influence on the free flap survival. Among 13 patients with complete final pedal arch after PTA procedure, there was no flap necrosis, while 4 flaps loss were found in the group of 10 patients with compromised pedal arch. Although, only one or two infrapopliteal arteries were often revascularized, we recommend that angioplasty should be attempted in all severe stenosis vessels, especially in triple-arterial stenosis case.

Hong has stated that evaluating perfusion of recipient arteries and identifying an appropriate vessel might be the major challenge in reconstructive microsurgery [3]. Previous studies have shown that an appropriate recipient artery could be selected preoperatively via angiography or CT angiography images [6, 22, 23]. Kim et al. have employed Doppler ultrasonography to check the recipient artery and demonstrated that peak blood flow velocity over 40 cm/s should be achieved to assure success in free tissue transfer [24]. Nevertheless, it has been indicated that pre-operation blood flow measurement might not be similar to actual finding in a surgery [3]. In our institute, we preferred CT angiography as a minimally invasive modality for vascular status assessment and flap planning. However, the final decision must be made based on intraoperative conditions of arteries. The surgery could be abandoned if sufficient vessels could not be found.

Based on evidence of safe utility of revascularized recipient artery, we also anastomosed flaps to 18 arteries that had undergone PTA [22, 25]. Although 3 of 18 flaps failed, we did not find significant correlation between the revascularized recipient artery and flap loss rate.

End-to-side anastomosis was performed in 40 flaps because it was believed that preservation of major arteries would be very important in CLI patients. In addition, flaps were also occasionally supplied by retrograde blood flow, especially in case of intact pedal arch. Lee et al. have found a significantly lower risk of wound complication of an end-to-side anastomosis rather than an end-to-end when they analyzed factors affecting flap survival and complication [9]. Although heavy calcification of arterial walls was common in PAD patients, we always tried to find the least atherosclerotic area for anastomosis. The end-to-end modality would only be used if we could not find less diseased area or unsatisfied result after end-to-side anastomosis.

There was no consensus in using fasciocutaneous or muscle flap combined with skin graft. We believe that fasciocutaneous is sufficient for diabetic foot ulcers that are often thin and superficial. In this study, ALT was the most common flap and MSAP flap was used as an alternative especially for forefoot and small defects. However, medial sural artery is considered as an important collateral around the knee, MSAP flap should be avoided in order to prevent further limb damage in patient with occlusion of popliteal artery [26, 27].

This study has several limitations including single-center retrospective design and small sample size, which restrict the ability of statistical analysis. Furthermore, the small number of flap fail in this study and the presence of several risk factors in a single patient also lead to some difficulty in determining the actual reason of flap failure. Further study with large size and is well designed is really necessary to make a power statement.

## Conclusions

In summary, our study indicates that the quality of pedal arch is crucial for flap survival in diabetic foot ulcer reconstruction. Thus, PTA should aim to re-establish a complete pedal arch to improve wound healing and flap survival.

## Data Availability

Please contact the corresponding author for data request.

## Abbreviations

PAD: Peripheral arterial disease
DM: Diabetes mellitus;
PTA: Percutaneous transluminal angioplasty
ESRD: End-stage renal disease
ATA: Anterior tibial artery
DPA: Dorsal pedis artery
PTa: Posterior tibial artery
ALT: Anterolateral thigh
MSAP: Medial sural artery perforator
